# The role of artificial intelligence for early warning systems: Status, applicability, guardrails, and ways forward

**DOI:** 10.1016/j.isci.2025.113689

**Published:** 2025-10-03

**Authors:** Timothy Tiggeloven, Samira Pfeiffer, Alessia Matanó, Marc van den Homberg, Lisa Thalheimer, Markus Reichstein, Silvia Torresan

**Affiliations:** 1 Institute for Environmental Studies, Vrije Universiteit Amsterdam, Amsterdam, the Netherlands; 2 CMCC Foundation, Euro-Mediterranean Center on Climate Change, Lecce, Italy; 3 United Nations University, Institute for Environment and Human Security UNU-EHS, Bonn, Germany; 4 510, an Initiative of the Netherlands Red Cross, The Hague, the Netherlands; 5 ITC/Faculty of Geo-Information Science and Earth Observation, University of Twente, Enschede, the Netherlands; 6 Population and Just Societies Program, International Institute for Applied Systems Analysis (IIASA), Laxenburg, Austria; 7 Department Biogeochemical Integration, Max-Planck-Institute for Biogeochemistry, Jena, Germany; 8 Department of Environmental Sciences, Informatics and Statistics, Ca’ Foscari University of Venice, Venice, Italy

**Keywords:** Earth sciences, Environmental science, Remote sensing, Artificial intelligence, Social sciences, Research methodology social sciences

## Abstract

Artificial intelligence (AI) is gaining momentum in earth sciences as a tool to analyze complex natural hazards and their impacts. Such analyses are critical for effective Early Warning Systems (EWSs), which is aiming to generate timely and actionable risk information to protect sectors, systems, and people. Despite advancements in AI, its role in EWS remains underexplored across the four pillars of the Early Warning for All (EW4All) framework; risk knowledge, forecasting, warning dissemination and communication and response preparedness. This study draws on a systematic literature review to assess AI methods utilized in the context of EWS, examines their challenges and opportunities and discusses guiding questions for responsible use. Our study highlights key gaps across knowledge, application and policy. Moreover, we call for coordinated efforts to develop responsible AI frameworks that enhance EWS while ensuring they remain inclusive, accessible, and people-centred that ultimately supports the goal of EW4All by 2027.

## Highlights

• Systematic review of AI utilization across the early warning chain
• AI in EWS shows exponential growth in papers published in recent years
• AI and EWS gaps exist across knowledge, application and policy
• Guardrails needed to ensure people-centred AI integration in EWS

## Graphical abstract

**Figure:**
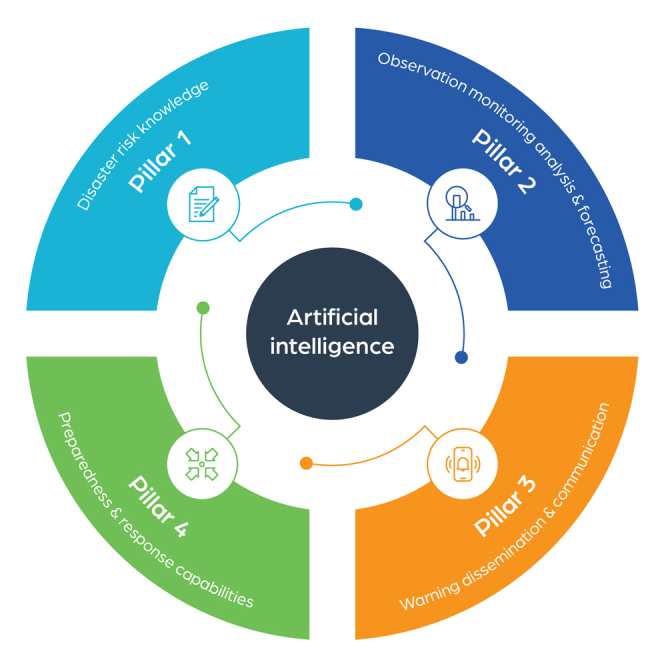

## Introduction

Artificial intelligence (AI) is key for the effectiveness of early warning systems (EWSs). However, ensuring that AI methods are used in a responsible and people-centred way requires advances in knowledge, application, and policy. In recent years, AI has rapidly transformed technological landscapes across sectors, offering unprecedented opportunities for addressing global challenges.^1^^,^^2^ Moreover, AI creates new avenues to improve the analysis of multidimensional data and accelerate information processing. This fast-developing field has improved how we enhance capabilities to handle large, non-linear, and complex datasets, simulate scenarios and aid decision-support mechanisms.^3^ While a range of sectors have employed AI, it has increasingly gained attention in the earth science and disaster risk reduction domain.^4^^,^^5^^,^^6^ Specifically, there is growing interest in using innovative methods such as deep learnings and natural language processes (NLPs) or technologies such as Internet of Things (IoT) in the context of EWS.^7^

EWS are recognized as one of the most effective tools for protecting lives, assets, and systems from hazards and their impacts.^8^ In contemporary frameworks, EWS encompass four interrelated pillars; risk knowledge (Pillar I), observation and forecasting (Pillar II), communication and dissemination (Pillar III), and response and preparedness (Pillar IV).^9^ As a substantial body of literature conceptualizes these four pillars as a people-centred framework which aims to ensure information and action reaches all relevant sectors and actors in sufficient time and leaves no one behind, it will guide this paper as conceptual understanding of the warning chain.^10^

The need for people-centred EWS across the four pillars is recognized in large scale global efforts such as the Early Warning for All Initiative (EW4ALL)—a United Nations (UN) program aiming to ensure everyone on Earth is protected by EWS by 2027—which responds to the Target G of the UN Sendai Framework 2015–2030 as one of the most effective instruments for reducing disaster risk.^11^

While the relevance of people-centred EWS for inclusive disaster risk reduction is recognized, half of the countries globally are not covered by EWS.^9^ Countries in the Global South and rural areas persistently report the largest gaps in terms of EWS coverage, while ineffective risk communication as well as a lack of operational preparedness plans are evident on all continents.^9^ This disparity is, however, illustrated by fundamental infrastructure deficits, such as Africa having just 37 weather radar stations compared to 636 in Europe and the United States for similar population sizes and landmass, and over 50% of existing stations provide data that is too inaccurate for reliable forecasting.^12^ Additionally, the digital divide may further limit the dissemination of warnings through for example inadequate internet access and warning messages that are not adapted to local languages, knowledge systems, or cultural contexts.

Furthermore, hazards are inherently complex^13^ and rarely occur in isolation.^14^ These so called multi-hazards can occur simultaneously or sequentially, interacting with the vulnerabilities and exposures of people, livelihoods, assets and systems,^15^^,^^16^^,^^17^^,^^18^^,^^19^^,^^20^ Here, we focus on EWS for hydrometeorological hazards and geohazards.^21^ Hydrometeorological hazards include both rapid-onset events like floods, hurricanes, and rainfall-triggered landslides with warning periods ranging from hours to days, and slow-developing events such as droughts with warning periods from weeks to months. However, EWSs for geohazards operate on widely varying timescales.^22^ For instance, earthquake operate on extremely compressed time frames, often mere seconds between detection and impact, while volcanic eruptions may exhibit precursor signals days to weeks before the events. Secondary hazards like tsunamis (triggered by earthquakes, volcanic eruptions, or landslides) and earthquake-induced landslides^22^ may further complicate multi-hazard warning frameworks, with each demanding specialized AI applications suited to their unique temporal scales and monitoring requirements. Furthermore, despite the critical need for addressing multiple hazards in a comprehensive manner,^23^ operational multi-hazard early warning systems (MHEWS) face challenges, with hazards typically addressed in isolation rather than accounting for their complex interactions.^24^ These complex interactions between and within risk components present significant challenges for most operational EWS, which monitor and assess single hazards only.

EWS present a potential domain for utilizing AI, along the whole early warning value chain.^25^^,^^26^^,^^27^ The emergence of AI capabilities has been enabled by concurrent advances in high-performance computing, massive datasets, modern algorithms, and high-level programming languages, creating a technological ecosystem more accessible to domain scientists.^28^^,^^29^^,^^30^ Recognizing this potential, the United Nations launched the Global Initiative on Resilience to Natural Hazards through AI Solutions, led by International Telecommunication Union (ITU), UN Environment Program (UNEP), UN Framework Convention on Climate Change (UNFCCC), Universal Postal Union (UPU), and World Meteorological Organization (WMO), which has been set up to ensure that advanced digital technologies will boost resilience to natural hazards.^26^ Supporting this broader vision, the global UN Early Warning Initiatives Executive Action Plan for EW4All (2023–2027) provides specific implementation pathways, while technical guidance on best practices for data collection, modeling and effective communication is emerging through ITU/WMO/UNEP Focus Group reports on AI for Natural Disaster Management and the subsequent Global Initiative on Resilience to Natural Hazards through AI Solutions that establishes frameworks for responsible AI integration across the four pillars. These initiative highlights that while physics-based prediction systems remain important, AI can complement them by reducing computational burdens, improving data processing efficiency, and enhancing predictions, especially where pure physical modeling is limited by process knowledge or computational capacity, e.g., for high resolution.^31^^,^^32^

In this study, we aim to examine potential gaps in the understanding and application of AI for EWS and provide evidence-informed questions that can help develop guardrails for responsible implementation of AI across the four pillars of early warning. We hypothesize three key areas requiring investigation.

(1) **The knowledge gap:** The current status and evolution of AI and EWS in terms of how adoption has progressed over time and geographical distribution is missing. Specifically, there are few studies that investigate the uptake of AI across the four pillars of early warning and the affiliated methods that have been used across different hazard types.
(2) **The application gap:** The use of AI across the four pillars is not yet well examined in a coherent way, and there is a lack of documentation of different AI methods for EWS by hazard types and a gap in examining the implications (opportunities and challenges) of utilizing AI across the four pillars of EWS (i.e., Pillar I-IV; risk knowledge, monitoring and forecasting, communication and dissemination and response preparedness). However, this is critical as it guides the ongoing efforts of people-centred EWS around the globe.
(3) **The policy gap:** There is a gap of studies that provide evidence-informed questions that can help to develop guardrails for research, practice, and policy for a responsible use of AI in the context of EWS (e.g., human oversight mechanism, ethical considerations). This can be a threat to the efforts to prioritize inclusivity and people-centred approaches (such as Target G of the Sendai Framework) to leave no one behind. Hence, it is critical to establish evidence-informed questions that can help develop context-specific guardrails for ensuring that “do no harm”.

Drawing on a systematic literature review, this paper presents, first, the results on the patterns of use of AI methods in existing early warning research, and the findings of the role of AI across the four pillars of EWS. Subsequently, we provide a reflection on cross-cutting challenges and opportunities while providing ways forward for addressing the knowledge, application, and policy gap. The insights of the paper are organized along the four pillars used in the EW4ALL initiative as a framework for implementation, to enable transfer of the results to practitioners and increase the relevance of findings for real-world application.

## Results

### Patterns of AI use and EWS

**Figure 1:**
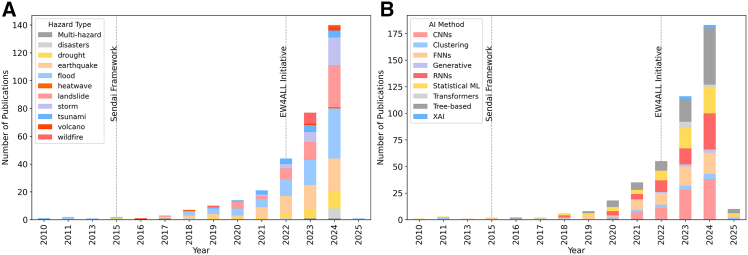
Distribution of scoped papers over time for hazard types and per methods Distribution of scoped papers over time for hazard types (left panel, A) and per methods (right panel, B). ANN, artificial neural network; CNN, convolutional neural network; LLM, large language model; NLP, natural language processing; RNN, recurrent neural network; ML, machine learning; XAI, eXplainable artificial intelligence.

Our systematic literature review revealed significant patterns in AI applications for EWS, illuminating knowledge, application, and policy gaps across different regions and throughout the four EWS pillars. Results show that, out of the total of 324 reviewed papers, a recent boom in literature on AI in the context of EWS coincides with the 2022 launch of the EW4ALL initiative^9^ (see Figure 1A). Multiple AI methods are applied, notably tree-based, CNNs, ANNs, and RNNs (Figure 1B). Furthermore we find that there is an exponential increase in papers between 2010 and 2025, with more than 40% published in the year 2024. Out of our final selection, we find that 55% of the papers are relevant to Pillar I, 38% to Pillar II, 6% to Pillar III and only 1% to Pillar IV. Figure 1 summarizes the distribution of scoped papers over time and by AI method utilised.

**Figure 2:**
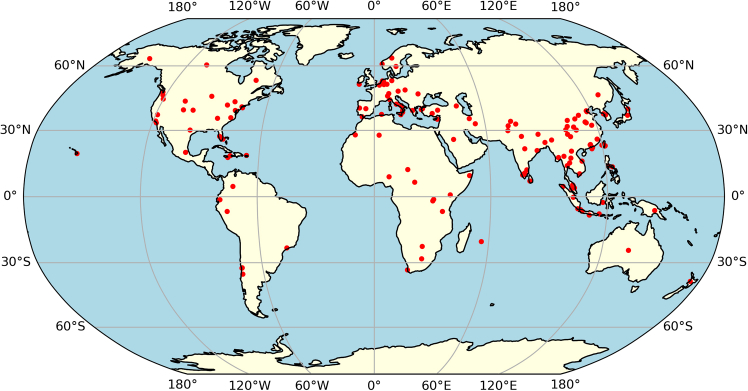
Distribution of study locations on AI methods for EWS Concentrations of cases are shown in USA, Europe, Southeast Asia, China, and Japan.

In total, we have found 158 specific locations mentioned in the reviewed papers as either location of deployment, monitoring or location of training data (purely research) (see Figure 2). The division is roughly 79% research-based case studies and 21% implemented or prototype. A focus on high-risk places where EWS are essential is highlighted by the fact that many case study locations are located along tectonic plate borders, along coastlines, and in areas that are prone to flooding. The spatial distribution of case studies reveals a strong concentration in Asia, Europe, and North America, with particularly dense clusters in China, India, Japan, and Southeast Asia. This may be due to the regions' high susceptibility to a variety of hazards, including earthquakes, landslides, floods, and typhoons. Western and Central Europe also show significant representation, which could be due to well-documented risk assessments and disaster response frameworks. In North America, most case studies are located in the eastern and western United States, aligning with research on hurricanes, wildfires, large coastal cities and flooding areas. In contrast, Africa and South America have comparatively fewer case studies, despite the vulnerabilities to hazards like droughts and floods in these regions, indicating potential research gaps. This geographical imbalance aligns with findings from UNDRR/WMO Global Status Reports on MHEWS highlighting similar disparities in early warning coverage and capabilities between resource-rich and resource-constrained regions.

Within the scoped papers of AI across temporal and geographical scales, there are typically three concepts used for EWS: (1) classical Machine Learning (ML), which involves algorithms that automatically improve their performance through exposure to data (such as clustering, regression, random forest, support vector machines); (2) Neural Networks (NNs), which is an advanced form of machine learning that utilizes interconnected layers of computational nodes to identify complex patterns within large datasets; and (3) Natural Language Processing (NLP), which extracts insights from unstructured text from reports, newspapers or social media (such as large language models). Note that while many studies in our review use the term Deep Learning (DL) to refer to NN with multiple hidden layers, we categorize all NN approaches, both shallow and deep architectures, under the broader NN category for clarity. A key difference between classical ML and NN is that in classical ML the user manually engineers the features/predictors, while in NN they are automatically learned from the data through the network’s layered structure extracted by the ML. Also called representation learning because good representations of the data are learned.^25^ More specifically, classical ML techniques like XGBoost and Random Forest can efficiently capture complex, non-linear relationships, and interactions within large, heterogeneous datasets, while NN excel at aggregating information across multiple dimensions, using CNNs to extract spatial patterns and RNNs or LSTMs to model temporal dynamics.

Most of the papers conceptually discuss applying either classical ML or NN methods for EWS related to floods (28%), earthquakes (21%) and landslides (17%), which also reflect the proposed maturity levels (implemented vs. prototype phase) of hazard emergency support by Merz et al.^33^ These events dominate across the four pillars of EWS with an exception for the reviewed papers that are linked to the response phase. Here drought-related studies are more prevalent. This suggests a stronger emphasis on using AI for real-time monitoring and preparedness for rapid-onset hazards, whereas slow-onset hazards like droughts receive more attention in post-disaster response and adaptation strategies.^34^^,^^35^^,^^36^ Notably, only four papers relate to multi-hazards, and even these do not directly use the term “multi-hazards” but rather address several hazards independently without implementing integrated warning systems, barring one on compound hazard interaction of ocean-fluvial floods. This highlights a significant gap in research specifically targeting the complex interactions between hazards that operational MHEWS would need to address.

Furthermore, the focus of AI methods varies by hazard type, as earthquake studies more dominantly apply NN, while drought-related studies almost exclusively use classical ML. This could be due to the nature of these hazards, as for example earthquakes involve complex spatial-temporal seismic and rock deformation patterns that benefit from deep learning’s feature extraction capabilities, whereas droughts, being slow-onset events, are often analyzed through structured decision trees that rely on threshold-based classifications.^37^^,^^38^^,^^39^ The distribution of methods among the pillars implies that each hazard’s unique characteristics have an impact on the model selection process. The relationship between hazard spatial-temporal scales and AI method selection is also evident: with real-time deep learning approaches often employed for rapid, localized hazards, while machine learning approaches for longer-term prediction and monitoring typically used for slow-onset, widespread hazards.

Table 1 provides a summary of various AI methods alongside examples of their applications for specific hazards.

**Table 1:** Artificial intelligence methods and use cases across EWS pillars for different hazards, with examples of applications of the 324 reviewed papers

AI method	Merits (or EWS application)	Hazard	Pillar	Examples of application	Reference(s)
**Linear/statistical ML methods** (e.g., logistic regression, naive Bayes, support vector machines, Bayesian, fuzzy logic), which provide baseline models but are less adaptable to complex interactions. They are efficient for binary and multi-class classification tasks.	they may serve as baseline models for hazard forecasting, offering rapid and understandable predictions that may support real-time decision-making.	flood	I	naive Bayes is used for flood susceptibility based on social media data in Chengdu city, China.	Li et al.^40^
		wildfire	I	SVM is used for predictive modeling of wildfires.	Sayad et al.^41^
		flood	II, III	fuzzy inference techniques are used for flood Impact-Based forecasting system.	Wee et al.^42^
		landslide	II	Bayesian Gaussian mixture model is used for automatic detection of rock-slope failures using distributed acoustic sensing.	Kang et al.^43^
**Clustering** (e.g., K-means, DBSCAN) algorithms group data points based on similarity without requiring pre-labeled outcomes, revealing inherent structure in the data.	they help identify spatial or temporal clusters of hazards, which may be valuable for the mapping of risk zones and targeted allocation of resources during emergencies.	flood	I	KNN are used to identify rainfall patterns for urban inundation rapid prediction.	Chen et al.^44^
		earthquake	II	K-means are used to label regional clusters which is feed into early warning detection of earthquake using deep learning.	Anggraini et al.^45^
**Tree-based methods** (e.g., random forests, decision trees, XGBoost) leverage ensembles of decision trees to capture nonlinear interactions and hierarchical relationships in data through recursive partitioning, making them robust for classification and regression tasks.	their robustness and interpretability make them ideal for identifying risk factors and generating classification or regression models for hazard prediction.	drought	II	XGB is used to predict Food-Security Crises in the Horn of Africa.	Busker et al.^46^
		tsunami	II	regression tree are used for tsunami waves forecasting.	Cesario et al.^47^
		drought	IV	fast and frugal trees are used for forecasting and unraveling early warning relationships between climate variability, vegetation coverage, and maize yields at multiple lead times.	Guimarães Nobre et al.^48^
**Feedforward neural networks (FNN)** consist of fully interconnected layers of neurons that learn complex nonlinear relationships through weighted connections and activation functions.	they integrate diverse data inputs to generate comprehensive risk assessments and predictions, which may support decision-making in various hazard scenarios.	tsunami	II	MLP is used for predicting the alert level due to a tsunami at given coastal locations.	De la Asunción^49^
		volcano	II	FCNN is used to classify the volcanic state of alert based on the behavior of certain features, providing a probability of having an eruption.	Rey-Devesa et al.^50^
**Convolutional neural networks (CNN)** are deep learning models that automatically learn spatial hierarchies from grid-like data using layers of convolutions.	**applications:** CNNs excel at analyzing remote sensing images to detect hazards such as wildfires, floods, and making them ideal for detecting seismic fields for earthquakes by extracting and interpreting spatial features.	earthquake	II	a CNN based architecture (PEGSNet) is applied to Instantaneously track earthquake growth with elastogravity signals.	Licciardi et al.^51^
		eildfire	II	U-Ccnvolutional long short-term memory (ULSTM) neural network is developed to extract the location and temporal wildfire evolution.	Bhowmik et al.^52^
**Recurrent neural networks (RNN)** are designed to process sequential data (such as time series data) by maintaining an internal state (memory), effectively capturing temporal dependencies.	their ability to model temporal dynamics makes them valuable for forecasting hazards, such as predicting weather patterns and event progression over time, and making them well-suited for hazards where historical patterns inform future risk.	flood	II	LSTM-based streamflow forecasting model is used to predict daily streamflow through a 7-day forecast horizon.	Nearing et al.^53^
		landslide	II	an LSTM-based model for early warning detection of landslide is developed using groundwater and rainfall monitoring.	Zhang et al.^54^
**Transformers** (e.g., large language models [LLMs] & natural language processing [NLP]) analyze and generate human language, enabling contextual understanding of large-scale textual data.	they extract actionable insights from disaster reports and social media, which facilitates early detection, human behavior or response of emerging hazards through text analysis.	wildfire	III	Dirichlet multinomial mixture (GSDMM) is used to detect trend and communication during wildfires.	Zander et al.^55^
		flood	II	ULMFiT is used as text classification to improve impact based weather warning systems and support decision-making.	Vrotsou et al.^56^
**Generative models** (e.g., autoencoders, GAN models), which reduce dimensionality and extract key features from high-dimensional data by learning efficient representations of the input.	they improve the quality of hazard datasets by filtering noise and highlighting critical patterns, thereby enhancing predictive accuracy in forecasting models.	earthquake	II	cascaded autoencoders are used for crowd detection and estimation for an earthquake EWS.	Lamas et al.^57^
		tsunami	II	encoder-decoder neural network is used for high resolution inundation mapping for tsunami early warning in Sicily.	Briseid Storrøsten et al.^58^

**Neural Network approaches** (such as CNNs and RNNs in Figure 1B), make up almost half of all applied techniques among all the other categories. Convolutional neural networks (CNNs), the most popular kind, are employed in 26% of studies (Figure 1B) and are primarily known for their ability to extract spatial features from images.^59^ In natural hazard research, they have been adapted to process structured time-series data, particularly for earthquake monitoring as seismic activity is often represented as spectrograms or spatial grids.^60^^,^^61^

Recurrent neural networks (RNNs) are another popular method, in particular for flood prediction, and are applied in 22% of the reviewed papers. RNNs are designed to handle sequential data, making them highly effective for hazards where historical patterns inform future risk. Specifically, Long Short-Term Memory (LSTM) networks, a type of RNN, are commonly used for flood forecasting and storm surge prediction as they retain information over long time intervals, capturing trends in atmospheric drivers, precipitation, river discharge, and soil moisture.^62^^,^^63^^,^^64^^,^^65^ Similarly, Feedforward Neural Networks (FNNs) (applied in 22% of the studies) are applied for flood and landslide prediction, providing flexible non-linear modeling but often requiring careful hyperparameter tuning and large datasets.^66^^,^^67^

Attention-based models reflect promising enhancement of EWS, such as through capturing complex spatial and temporal dependencies in environmental data. Recent advances like Earthformer demonstrate how AI architectures can process large-scale spatiotemporal data more efficiently and that subsequently enables faster extreme weather predictions across broader geographic areas, which is a critical improvement for extending early warning lead times.^68^ This architecture has demonstrated state-of-the-art performance in Earth system forecasting tasks, including precipitation nowcasting and El Niño/Southern Oscillation (ENSO) forecasting. Another example is Contextformer, which is introduced by Benson et al.,^31^ and is a multi-modal Transformer model designed for high-resolution vegetation forecasting. By integrating spatial context through a vision backbone and modeling temporal dynamics with meteorological time series, Contextformer effectively predicts vegetation greenness across Europe, which is another important indicator for several hazard predictions. These attention-based models have been applied to various EWS. For instance, an attention-based temporal CNN has been proposed for predicting landslide warning signals, demonstrating high accuracy in capturing precursory warning characteristics from sensor data.^69^ Additionally, attention mechanisms have been incorporated into CNN to improve flash flood susceptibility modeling, as shown in studies focusing on ungauged watersheds.^70^

**Classical ML methods** for EWS, and specifically tree-based approaches, are applied in 32% of the studies (Figure 1B). These approaches include Random Forest (RF) and Gradient Boosting Machines (GBMs), which are particularly useful for EWS as they can provide interpretable decision-making processes, and can handle non-linearity and missing data effectively.^71^ Tree-based models excel at capturing relationships between environmental indicators and hazard occurrence, making them well-suited for hazards with gradual development, such as droughts and landslides.^72^^,^^73^

**Explainable AI** (XAI) methods are also represented in few studies, notably to improve transparency in EWS models and these XAI techniques, such as SHAP (Shapley Additive Explanations) and LIME (Local Interpretable Model-Agnostic Explanations), help interpret classical ML and complex NN models by highlighting feature importance and decision pathways.^74^^,^^75^ In the realm of hazard forecasting and EWS, this may be particularly useful as trust and interpretability are crucial for decision-making.^76^

### Role of AI across the four pillars of early warning

AI methods provide merits for the application across the four pillars of early warning-while also posing challenges for different use cases along the warning chain.

#### Disaster risk knowledge (Pillar I)

Building risk knowledge is the basis of EWS because it enables more accurate predictions, timely communication, and effective preparedness measures.^8^^,^^77^ In recent years by using AI, significant progress has been made to better understand the complex relationships between hazards, society, and risks and conduct risk assessments.^75^^,^^78^^,^^79^^,^^80^ A common first step is establishing a shared glossary of terms to ensure consistent understanding across disciplines, as exemplified by the ITU-T Focus Group Technical Report on Artificial Intelligence for Natural Disaster Management, which provides standardized definitions within the *trans*-disciplinary domain of AI for risk management. This study adopts and builds upon these established terminologies.

Risk knowledge for EWS should encompass and integrate the concepts of hazard, exposure, vulnerability, and impact-based forecasting, however few of the reviewed papers explicitly address these dimensions alongside hazard prediction and most of these studies focus on drought and flood as the main hazards. Shyrokaya et al.^73^ demonstrate that integrating exposure and vulnerability for impact-based forecasting—using fuzzy inference, machine learning, and multi-source data fusion—has enabled more precise, lead-time predictions and actionable risk communication in EWS. However, significant challenges remain in normalizing exposure and vulnerability metrics to account for dynamics,^73^ addressing data gaps,^81^ and incorporating dynamic socio-economic factors.^38^^,^^42^^,^^73^^,^^82^ These challenges present opportunities for further innovation in model refinement, enhanced data integration (e.g., from stakeholders and local and regional assessment documents) into adaptive forecasting frameworks.^56^ The integration of these dimensions into risk frameworks is especially critical given that advanced data-driven methods, including AI, demand large training datasets—yet we face a substantial shortage of reliable data on impact and vulnerability metrics.

At the same time, multi-hazard and impact-based approaches supported by AI remain underrepresented, despite a few promising studies.^15^^,^^83^ However, as disasters are complex and interconnected, developing models that account for multiple interacting hazards—rather than isolated ones—will be crucial to building a resilient and adaptive early warning framework that reflects the complexity of real-world risks.^17^^,^^84^^,^^85^ One of the challenges for MHEWS would be to standardize evaluation metrics tailored to specific hazards—such as floods, earthquakes, and landslides—a method that could be applied by various AI models and by doing so will also foster better model validation and cross-comparison.^86^^,^^87^

Employing generative adversarial networks (GANs) to create synthetic datasets has shown considerable potential for improving landslide displacement models, while federated learning approaches enable the aggregation of localized models—such as those used in earthquake prediction—without compromising sensitive data.^88^^,^^89^^,^^90^ These federated approaches enable multiple institutions to collaboratively train shared AI models by exchanging only model updates rather than raw data, which makes them particularly valuable for contexts with sensitive data or limited connectivity where traditional centralized approaches are not feasible due to for example data constraints. Additionally, exposure, vulnerability and impact datasets can be derived from AI. For example, for exposure mapping, there are promising approaches that include building footprint extraction using models like Google Open Buildings, Microsoft Bing Maps Building Footprints, and AI-enhanced population distributions from WorldPop that include poverty indicators.^91^ Physical vulnerability assessments can, for example, leverage pre-labelled damage datasets like xBD (which contains approximately 850,000 labeled damages across multiple hazards) to train CNN models that assess building damage for new disasters or establish baseline vulnerability.^92^ Subsequently, CNNs can be used to determine the damage for an unseen disaster, forming an alternative rapid damage assessment, but also to train impact-based forecasting models if other impact data are missing.^93^ Additionally, the use of NLP for enhancing the collection of impact data has proving promising^94^ and the same method could be applied to gather information on early actions and responses to supplement traditional impact records.

Foundational models like large-scale Earth system models and multimodal AI systems can enhance disaster risk knowledge (Pillar 1) by synthesizing vast amounts of environmental observations and extracting complex spatiotemporal patterns that may inform a comprehensive understanding of Earth systems.^95^^,^^96^^,^^97^ These models, for example, are addressing the challenge of integrating heterogeneous observation networks and models to improve predictions across scales from weather to climate. Recent developments such as Aurora-based on more than a million hours of geophysical data show that such models can outperform traditional numerical forecasting systems across multiple Earth system domains and are orders of magnitude more computationally efficient, allowing for the enabling broader accessibility to accurate environmental predictions underpinning effective EWS.^98^

These strategies enhance model robustness and democratize access to high-quality risk assessments, paving the way for more resilient and community-focused EWS.^99^ However, addressing data scarcity through innovative methods like AI-derived datasets may fill data gaps but not change the issue of data scarcity as such.^100^^,^^101^^,^^102^^,^^103^^,^^104^ For example, global gridded population datasets systematically underrepresent the rural population-which is not necessarily changed through AI analysis methods.^105^

#### Monitoring, forecasting, analysis of hazards (Pillar II)

The WMO Executive Action Plan for EW4All (2023–2027) emphasizes that enhanced data integration and technological innovation are a core priority to strengthen monitoring and forecasting capabilities. AI is progressively transforming Pillar II, enhancing the monitoring, analysis, and forecasting of hazards through its ability to process vast amounts of real-time data from diverse sources.^37^^,^^43^^,^^106^ Techniques like deep learning, transfer learning, and hybrid models drive these significant improvements in prediction accuracy, lead times, and location determination, as is demonstrated by studies such as those by Abdalzaher et al.^107^^,^^108^ and Xu and Gao.^109^ A recent innovative advancement within AI for forecasting is GraphCast, which provides a graph neural network-based forecasting system that delivers highly accurate, medium-range weather predictions and early warnings of extreme events.^32^ The potential of transformative AI models that include LLMs and foundation models may enhance disaster risk management across multiple domains and is comprehensively examined in the ITU-T Focus Group Technical Report on Transformative AI Models for Natural Disaster Management, which provides guidance on leveraging these advanced AI architectures for improved monitoring and forecasting capabilities. Additional method advancements center around integrating AI with real-time sensor networks, satellite data, and IoT to create adaptive, intelligent systems that can detect weak hazard signals earlier than traditional models, crucial for improving global early warnings.^52^^,^^110^ This shift toward dynamic and data-driven forecasting is marking a key advancement in global risk management and reduction.

To successfully integrate AI in Pillar II, the integrity and reliability of data streams from heterogeneous sources is important. For example, by ensuring data quality, robust sensor calibration, and secure communication networks, models may be able to avoid false alarms or missed events—a concern highlighted by Li et al.^111^ and Al-Rawas et al.^112^ in their review of flash flood prediction technologies. Similarly, standardized interoperability protocols to seamlessly integrate IoT sensor data, satellite imagery, and numerical model outputs, would provide guardrails for monitoring and detection as these elements form the backbone of efficient EWS.^108^^,^^113^^,^^114^ Zhu et al.^115^ demonstrate how AI can automatize autonomous location-based decision-making to transform and enhance emergency response operations. Furthermore, standardizing protocols in the form of data-knowledge-driven or collaborative frameworks, as those proposed by WMO, is holding the key to transforming proactive hazard mitigation in the face of a rapidly changing climate.^52^^,^^116^

Processing these multi-modal data in near real-time with the help of advanced AI models is a substantial opportunity for enhancing hazard forecasting. For instance, deep learning architectures that are integrated with autoencoders and CNNs, can rapidly estimate earthquake parameters and thereby improving early warning lead times.^108^ Furthermore, Xu and Gao^109^ show that high accuracy and low computational cost can be achieved by developing a hybrid surrogate model that fuses LSTM and CNN outputs, which they exemplify with a coastal flood prediction study. Furthermore, investing in distributed sensor networks with edge computing capabilities could be worthwhile as these methods enable local data processing to lower latency and improve the promptness of warnings. Such integration enables AI to process high-speed data streams and detect subtle, weak hazard signals much earlier than conventional statistical models, thereby extending the effective lead time of EWS.

Another promising avenue is enhancing the generalisability of AI models across different hazards and geographical regions that can be done through transfer learning and multi-modelling. For example, deep learning architectures can be adapted to diverse environmental conditions, while maintaining high prediction accuracy despite variations in regional hazard characteristics.^117^^,^^118^ This adaptability not only facilitates the deployment of robust EWS in data-scarce regions^102^ but also opens possibilities for a unified forecasting framework that can address multiple hazard types simultaneously.^119^ However, the integration of multi-hazards remains challenging as different hazards have different lead times requiring different ways of operating.^22^ Developing flexible, modular AI architectures that are able to accommodate variable temporal scales and warning thresholds, while maintaining interoperability between hazard-specific components, will be critical to overcome these operational challenges and enable to adapt to changing environmental conditions.

Learning algorithms that can quantify uncertainties in hazard forecasts-such as Bayesian AI techniques and traditional physics-based models-can lead to more robust and interpretable predictions.^109^^,^^118^^,^^120^ For example, models that integrate physics-based with advanced AI models can give insights into the fundamental dynamics of hazard processes.^121^^,^^122^^,^^123^ A prime example of an integrating all approach, is establishing a digital twin that is able help precise risk prediction while leveraging AI algorithms for efficient processing and analysis of real-time data.^75^ Digital twins allow for the integration of real-time sensor data, advanced simulation models, and historical records into a unified virtual replica of physical systems, enabling continuous monitoring, dynamic risk assessment that include exposure and vulnerabilities of the system, and proactive disaster management.^124^^,^^125^^,^^126^^,^^127^^,^^128^ Multi-hazard susceptibility maps may provide a foundation here from which to capture and contextualize underlying environmental and atmospheric processes, as well as hazard interactions.

However, to provide actionable risk information that enables targeted protective actions it is crucial to integrate exposure and vulnerability data with hazard intensity warnings.^129^^,^^130^^,^^131^ We acknowledge, however, that dynamic exposure and vulnerability data are generally scarce and difficult to collect,^132^^,^^133^ particularly for the most vulnerable groups (e.g., residents of informal settlements). When exposure and vulnerability data do exist, for example, collected by insurance companies and usually in high-income countries, they are often not publicly accessible or incomplete, Potential pathways to overcome these limitations include enhancing, public-private partnerships, anonymized data sharing agreements, and the development of standardized vulnerability indicators from open government data sources. Additionally, the use of crowd-sourced vulnerability mapping or the integration of satellite-derived exposure metrics offer alternative approaches to complement unavailable data. These impact-based forecasts represent a strategic priority within the EW4All initiative, which allows to shift from traditional hazard-only predictions to forecasts that directly estimate potential consequences on people, infrastructure, and livelihoods. AI may play a crucial role in impact-based forecasting as it allows for the processing of complex multi-dimensional datasets that combine meteorological predictions with demographic, infrastructure, and socioeconomic data to generate location-specific impact predictions.^25^^,^^134^ For example, instead of simply forecasting flood depth, impact-based systems allow for predictions that can warn communities that they will be cut off, or provide information on which critical infrastructure will fail, and what humanitarian needs will emerge.^135^ This transformation from ‘what the weather will do’ to ‘what the weather will due to us’ is essential for moving beyond generic warnings to tailored, actionable guidance that saves more lives and reduces losses.^136^

#### Warning dissemination and communication (Pillar III)

Enabling clear and accessible dissemination and communication is crucial for the translation of early warning information into actionable formats. For example, the common alerting protocol, Google Public Alerts, and IFRC Alert Hub expand the reach of reliable, fast, and actionable warning messages to people at risk, in which AI could support. Moreover, operational guidance from international organisations is emphasizing the critical role of AI to enhance communication effectiveness. For example, the WMO Guidelines on ‘Multi-Hazard Impact-based Forecast and Warning Services’ highlight the importance of partnerships between scientists, forecasters, and community leaders in developing effective warning communication systems.^137^ Furthermore, the ITU/WMO/UNEP Focus Group on AI for Natural Disaster Management (FG-AI4NDM) has established best practices for using AI to support EWS and improve communication across spatiotemporal scales through multiple operational use cases how AI-enhanced communication systems can improve alert dissemination across diverse populations.^138^

First, AI can generate emergency alerts tailored to specific geographic locations, demographics, and language preferences. The use of large language models to support the translation of warnings is, for instance, a straightforward application and has already been implemented in several operational EWS. However, more work is needed in tailoring messages to recipients based on their demographic characteristics. While progress has been made in creating sector-specific warnings, it is essential to further develop personalized alerts, for example, for people of different ages or persons with disabilities. Core topics in this area include real-time alert generation, explainable alerts, and the development of user-friendly and accessible communication interfaces, which together facilitate effective disaster risk communication and situational awareness.

Secondly, another area of development is the AI-driven real-time prediction that can quickly generate alert messages. For instance, Dang et al.^139^ developed a real-time EWS for urban flooding that is leveraging big data analytics and Web-GIS visualisations that is able to enhance flood risk communication. Another example is “FloodWatch,” which is an IoT-based flood monitoring system that provides continuous hazard assessment and instant notifications.^140^ Furthermore, Ouaissa et al.^141^ highlights the role of AI and IoT integration in wildfire and flood management, demonstrating how real-time processing improves situational awareness and response capabilities of people at risk. Furthermore, AI can integrate user reports from social media and IoT devices, providing a comprehensive situational overview in real time, which increases the effectiveness of information dissemination to end-users^34^ For example, leveraging the collaborative power of AI and citizen science can be complementary by improving the use and access to citizen generated data, which supports inclusion of complementary local knowledge to forecast models.^133^ These approaches are aligning with findings from the IFRC’s guide ‘The Future of Forecasts’ that is demonstrating how impact-based forecasting can transform complex scientific information into actionable community insights for (AI-powered) communication strategies.

Third, AI can support by real-time analysis of multi-modal data—from sensor networks, satellite imagery, radar, weather models, and social media—to generate timely, actionable warnings and ensure that emergency messages reach all stakeholders via intuitive digital platforms. For the public, it is essential to provide intuitive and multi-platform communication strategies. If interfaces are able to provide alerts that are understandable through web platforms, mobile applications, and interactive mapping tools, it may enhance public engagement.^139^^,^^140^ For example, visual and geospatial representations of risks, such as real-time hazard mapping and augmented reality overlays, make complex data more comprehensible for both decision-makers and communities. An underrepresented aspect is the use of AI for reinforcement learning-training agents for decision-making during emergency scenarios, which could be an area of future research.

#### Preparedness and response capabilities (Pillar IV)

Enhancing response capacities and preparedness is essential for ensuring that EWS translate into timely, effective actions that minimize the societal and economic impacts of natural hazards. AI is progressively employed to test its effectiveness in supporting pillar IV, notably in modeling disaster response scenarios, speeding up analytics and data processing for real-time relief efforts, and improving the efficiency of emergency preparedness across sectors and decision-making support systems with diverse stakeholders connected to EWS and anticipatory action.^142^^,^^143^ While most operational EWS have not yet fully integrated AI, existing humanitarian frameworks may provide valuable foundations for AI implementation to support. For example, the IFRC’s Operational Framework for Anticipatory Action 2021–2025 establishes systematic approaches for forecast-based disaster response that could be enhanced through AI applications in risk assessment, resource allocation optimization, and predictive modeling. OCHA’s briefing note on AI for the humanitarian sector reflects this as well, although mentioning some challenges to overcome in a data driven world, such as challenges misinformation, reinforcement of bias, system opacity, cybersecurity, and erosion of privacy.^144^ Additionally, to ensure a coordinated progress in AI-enhanced disaster risk management technologies for preparedness and response capabilities across international organisations and standards development bodies, the FG-AI4NDM provided a standardization roadmap, which may serve as a strategic guide for this.^145^

A range of examples of AI-support in this domain come from humanitarian and military sectors, such as using unmanned aerial vehicles (UAVs) to capture and process high-resolution local real-time data in emergency scenarios, such as AI-supported spatial mapping, data processing for damage assessment and situational awareness. On the financing side, Fast and frugal tree methods could be used to analyze the existing rapid cash transferring systems in a forecast model that unravels early warning relationships between climate variability, vegetation coverage, and maize yields at multiple lead times and cost-effectiveness of response measures.^48^ Additionally, lessons can be drawn for AI-based responses to natural hazards from other field such as the health sector and biological hazards. For example, AI is used to simulate COVID-19 vaccine delivery contingency plan for IDP camps in Borno State, Northeast Nigeria. Next to this, AI methods have supported simulating the transmission of infectious diseases under various intervention measures and evaluate the effectiveness of control strategies can help formulate, implement, and potentially adjust measures.^146^

Despite these promising applications, multiple challenges exist, such as uncertainties and the algorithm bias, might lead to false response scenario planning, notably by reinforcing the underrepresentation of rural communities and minority groups.^132^ Managing the complex interrelations of disaster response scenarios remains challenging as new risks could be introduced while advanced technology may lack applicability or fail to meet the actual needs of rural communities.^147^^,^^148^ While in existing systems, this is already a challenge, automated models may narrow this people-centric interaction along the warning chain even more.

With increasing complexity and predictive power of AI models, issues of trust, reliability, and interpretability of the models and its suggestions are fragile. This can cause issues from a physical perspective but can also result in a lack of trust by multi-stakeholders in warning messages.^76^ Transparency in AI systems extends beyond mere technical openness; it involves providing stakeholders with accessible and meaningful information about how AI models function, make decisions, and impact various user groups, ensuring that AI within the warning chain is transparent and inclusive.^149^

#### Cross-cutting aspects of responsible AI in EWS

While AI offers significant potential across all four pillars, successful implementation requires to address fundamental challenges that may transcend individual pillar boundaries. Here we identify four critical cross-cutting aspects. Firstly, explainability is critical-yet, challenging for ethical and inclusive usability across the four pillars.^150^ For example, to ensure that AI-driven early warning communications are actionable, it is essential to have explainable results that preferably also provide insides on uncertainty. The lack of interpretability of AI can hinder trust in AI-generated warnings, making it essential to incorporate explainable AI (XAI) methods that reveal decision-making processes and highlight uncertainties.^76^ For the interoperability of EWS, where human lives depend on, it is crucial to provide such transparency and insights into model predictions. Ultimately, this will enhance user confidence also contribute to better-informed decision-making in high-stakes hazard scenarios.^75^^,^^79^

Secondly, accountability is a key need in AI-powered EWS, notably, to establish clear responsibility frameworks for when systems fail, which ensures humans retain ultimate oversight of warning decisions regardless of automation level.^150^^,^^151^ For example, transparent decision-making chains must document, which components (AI or human) triggered specific warnings, which should enable post-event auditing and continuous improvement while clarifying liability. Additionally, accountability demands for inclusive governance structures where diverse stakeholders—including vulnerable communities—have meaningful input into system design, deployment, and evaluation, ensuring AI serves broad public interests rather than narrow technical or commercial goals. Furthermore, collaborative approaches are key to evolving EWS.^77^ For example, fostering interdisciplinary collaborations among geoscientists, AI experts, policymakers, and local stakeholders is essential to translate these technological advances into sustainable, real-world applications.^152^

Thirdly, data scarcity in AI-powered EWS reflects insufficient historical records of extreme events, inadequate monitoring networks in vulnerable regions, and low-resolution datasets of hazard-specific parameters.^153^ Such scant training data can produce biased models that perform poorly in historically underrepresented areas or for rare but catastrophic events and thus create warning gaps between data-rich and data-poor regions. Beyond simple data augmentation, novel approaches are needed for addressing this challenge: physics-informed models incorporating domain knowledge, transfer learning from data-rich to data-poor contexts, and methods to quantify uncertainty when working with limited observations. Addressing data gaps in training data for data scarce regions, is to prioritize the development and validation of AI-derived datasets across risk components. AI-derived datasets should be rigorously validated with ground-truth observations where available, with clear documentation of uncertainties, potential biases (especially in underrepresented regions), and methodological limitations. International standards for dataset quality, interoperability, and transparency would further enhance the utility of these resources for operational MHEWS, particularly in data-scarce regions where traditional observational networks remain limited. The ITU/WMO/UNEP Focus Group on AI for Natural Disaster Management and the subsequent Global Initiative on Resilience to Natural Hazards through AI Solutions provide technical guidance on best practices for data collection, modeling and effective communication for addressing these standardization challenges for responsible AI deployment that could help bridge data and capability gaps across regions.

Fourthly, community engagement and local knowledge integration should be emerging as critical success factors that will determine AI-enhanced EWS effectiveness. The IFRC’s extensive experience with forecast-based financing across multiple National Societies is demonstrating that meaningful community engagement is essential for any EWS to be trusted, understood, and acted upon. Successful early warning implementations should have a systematic way to integrate traditional and Indigenous knowledge systems alongside technological solutions, which is documented in Anticipation Hub case studies.^154^ Additionally, organisations such as Practical Action exemplify usage of AI in case studies, while UNDRR point out the potential of AI for EWS and documents this in their handbook on risk knowledge for MHEWS. This would establish principles that will be vital for ensuring that AI systems complement rather than replace local expertise and decision-making processes. Additionally, co-production approaches are essential for successful AI implementation in EWS and should involve meteorologists, social scientists, and humanitarian experts that work directly with at-risk communities,^155^^,^^156^ as is emphasized by the IFRC’s comprehensive analysis in ‘The Future of Forecasts’ and WMO’s guidelines on ‘Multi-hazard Impact-based Forecast and Warning Services’.

## Discussion

### Ways forward

**Figure 3:**
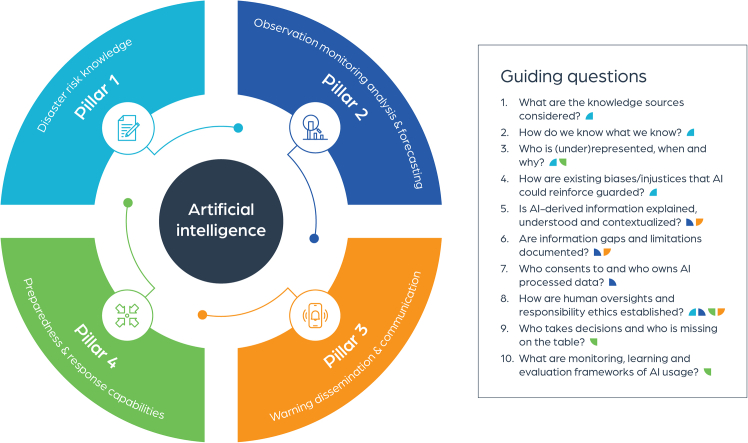
Role of guiding questions that serve as guardrails for the responsible utilization of AI methods across the four pillars of EWS The EW4ALL framework is adapted from WMO’s Executive Action Plan for 2023–2027.

This study aims to address the knowledge, application, and policy gap on AI and EWS across the four pillars. While AI holds significant potential to improve EWS across these pillars, our results highlight the necessity of establishing guardrails for responsible use of AI in EWS, to ensure people-centred approaches and address the current challenges emerging in use cases (such as data gaps, algorithm bias, and underrepresentation of minority groups). While this study did not aim to establish universal guardrails for AI and EW, it intended to provide evidence-informed questions that need to be considered and addressed by those who have the responsibility to implement and operate EWS. Hence, Figure 3 provides an overview of guiding questions based on this study that can help to establish guardrails across the four pillars to contribute and transfer to current knowledge, implementation, and policy sphere of EWS and AI (e.g., under the EW4ALL initiative). It unravels some of the key issues that are critical for responsible use of AI methods across the four Pillar, notably to ensure people-centred, responsible, and accountable AI use. The guiding questions are not comprehensive, nor extensive, however, they offer a guiding set of questions for future research in this domain moving forward.

#### The knowledge gap

Our review finds that AI can support in integrating exposure, vulnerability, and impact-based forecasting by standardizing metrics, addressing data gaps or hybrid approaches that combine physics-based models with advanced AI. For example, digital twins and collaborative systems may offer promising pathways to enhance dynamic risk prediction and proactive disaster management. While standardizing evaluation metrics across diverse hazards might remain a significant challenge, developing models that capture and that quantify the complex interactions of cascading events might offer an interesting array for future research. The review also highlighted that one of the major trends in AI and EWS are methods that can help to address data scarcity, specifically in rural areas (data augmentation, transfer learning and federated learning to overcome data limitations) and enhance model robustness and democratize access to high-quality risk assessments for EWS. Filling those data gaps, however, it will be critical to consider diverse knowledge sources and reflect on the biases of the data and algorithms utilized (see guiding question 1–3). Specifically, the narrative of filling data gaps through AI needs careful reflection on accountable oversight mechanisms to ensure quality and reliability (see guiding question 7–9).

#### The application gap

AI has been increasingly applied in the domains of risk knowledge, monitoring and forecasting, dissemination and communication and preparedness and response. However, the main application area remains in monitoring and forecasting. Another finding of current applications is the processing of multi-modal data in near real-time using advanced AI architectures and distributed sensor networks. Specifically, AI has the potential to reduce latency and extend early warning lead times accuracy in monitoring and forecasting. Further, its capacity to model generalisability through transfer learning and multi-modelling paves the way for improving forecasting across diverse hazards and support scenario planning. Finally, integrating physical process simulations with data-driven insights or AI powered earth system models and digital twins hold promising pathways for developing robust, interpretable systems that better inform timely decision-making. However, it is impediment to establish evaluation criteria ensuring data integrity, accountability, and interoperability across heterogeneous sources—such as IoT sensors, satellite imagery, and numerical models (see guiding question 10). This can mitigate false alarms and improve detection reliability. Additionally, the application areas of AI are dominantly technical niche-requiring efforts for explainability and ensuring that the role of AI in the warning chain is well documented and understood by all stakeholders (see guiding question 5 & 6). Furthermore, we showed that case studies are mostly absent in sub-Saharan Africa, Central America, parts of South America, Central Asia, and small island developing states. Such uneven geographical distribution of AI-focused EWS research and implementation reflects the infrastructure and digital disparities, where regions most affected by the digital divide and weather station deficits receive the least research attention, creating a worrying cycle where technological advances may actually worsen rather than reduce existing warning system inequalities (see guiding question 6 & 9). To bridge this digital divide, Digital Public Goods initiatives and open-access Earth observation platforms such as Digital Earth Africa and Copernicus are offering promising pathways by providing free access to satellite data and computational resources. This has the ability to support and enable researchers and practitioners in data-scarce regions to develop locally relevant AI-EWS applications.

#### The policy gap

While advanced AI methods can enhance the effectiveness across the warning chain, it persists a lack of studies providing ethical and human-rights based frameworks and commonly agreed ethical oversight mechanism for AI deployment (see guiding question 8). For example, few of the studies applying AI methods in EWS domain, provide reflection and ethical guidance on issues such as privacy, data bias and the trustworthiness of AI-supported information for policy makers and practitioners. Global, regional and national standards need to be developed for the use of AI in EWS, building upon existing frameworks such as the ITU/WMO/UNEP technical reports on AI for Natural Disaster Management and the WMO Executive Action Plan implementation guidelines.^157^ For example, the protection of transparency, fairness and human oversight of AI systems are impediment for implementation in any EWS application (see guiding question 8). Specifically, core values such as gender, education, research, wellbeing and ecosystems need to be integrated, evaluated and monitored through frameworks and policy mechanism for AI in EWS, while currently critically underrepresented or even absent^72^ (see guiding question 3).

The scope of this study was not aimed at establishing comprehensive guardrails for addressing such policy issues. However, it recognizes that technocratic approaches in disaster risk reduction must go hand in hand with people-centred and responsible approaches, which are yet to be established for this domain. Guardrails are almost absent in the reviewed papers in this study. Additionally, it is essential to foster international standardization efforts that include diverse stakeholders representing different regional priorities. This ensures both effective and equitable implementation, potentially encouraging adoption of these standards into national legislative frameworks.^158^ Further, such collaboration allows to develop international standards for AI in EWS that are not limited by cross-border interoperability, which are currently critical blind spots in warning coverage precisely where hazards may transcend national boundaries. Ultimately, unlocking the potential of AI in EWS requires close collaboration between the research community and the key national actors -supported by the UN agencies and IFRC- implementing EW4All, including UNDRR for risk knowledge and preparedness, WMO for monitoring and forecasting systems, ITU for warning dissemination and communication and IFRC for preparedness to respond. Such an alignment would ensure that research priorities identified here translate into operational improvements across the four pillars and support evidence-based policy development at international and national levels. The guiding questions that serve as guardrails for responsible AI implementation and pillar-specific findings from this review offer these agencies a practical framework that can be used to develop responsible AI integration strategies that can address the technical, ethical, and equity challenges identified across EW4All implementation.

### Concluding remarks

This study examined AI methods utilized in the context of EWS, their opportunities and limitations and discusses guardrails for applying AI in people-centred EWS. While AI offers opportunities for the effectiveness of EWS, there is a lack of guidance and ethical questions to ensure inclusive, people-centred warning systems moving forward. Artificial intelligence (AI) paves the way for improvements of early warning across the four pillars, supporting its viability for protecting sectors, systems, and people.

*Patterns of AI use/adoption*: AI tools in EWS substantially increased in the past decade across the globe for different hazard types in the domain of early warning. A variety of methods are utilized (such as Deep Learning, NLP) to support parts of the warning chain, dominantly computational and data-driven components.

*Challenges and opportunities across the four pillars:* Application areas are dominantly in forecasting and monitoring, however, there are emerging patterns and examples for applying it across the warning chain, which need to be further explored. Challenges include explainability, including privacy and ethical considerations of the use of data, issues around accuracy and accountability of AI and the data scarcity challenge.

*Addressing policy, research, and knowledge gap:* The role in EWS is not a silver bullet for improving existing systems-it can be understood as a complementary approach, when carefully tested and ethically embedded into regulatory frameworks, to support parts of the warning chain. It is critical in the future to strengthen research and policy making of people-centred EWS and DRR to identify utilities that do no harm.

Future research can strengthen the responsible use of AI methods across the EWS pillars through tackling guardrails and providing guidelines for research, application, and practice. Ultimately, the potential of AI in EWS could only be realized with close collaboration between the research community and UN agencies implementing EW4All (including WMO, UNDRR, and other relevant agencies) to ensure that research findings would be translated into operational improvements and evidence-based policy development across the four pillars.

### Limitations of the study

This study encounters multiple limitations in the methodology. First, we acknowledge that there are other papers that still address AI for one of the EWS pillar but not explicitly mention early warning or early warning systems in their paper. For example, there are papers that are about preparedness in humanitarian action, which might have also contributed additional insights, however, due to the selected search strings were not integrated. Secondly, the four-eye principle of title and abstract screening might have introduced biases due to the positionalities of the two researchers for selecting the papers. Thirdly, only including open access and English papers, as well as excluding gray literature in the systematic literature review introduces limitations to the comprehensiveness of the review. Fourthly, the study utilized NLP in the review process to extract locations or hazard types of the reviewed abstracts, which introduces the bias of an AI tool to the research; however, 100% human oversight was implemented to mitigate this limitation. Lastly, the requirement for explicit 'early warning' terminology in our search may have excluded relevant AI forecasting studies using alternative terms such as forecasting and prediction in the realm of hazard studies that might be of interest to one of the pillars of EW4ALL.

## Resource availability

### Lead contact

Requests for further information and resources should be directed to and will be fulfilled by the lead contact, Timothy Tiggeloven (timothy.tiggeloven@vu.nl).

### Materials availability

The study did not generate new materials.

### Data and code availability

• Data on the reviewed papers in the systematic review are available in the supplemental information. All other data reported in this paper will be shared by the lead contact upon request.
• The code related to this article can be accessed by reaching out to the lead contact, provided the request is reasonable.
• Any additional information required to reanalyze the data reported in this paper is available from the lead contact upon request.

## Acknowledgments

T.T. and S.T. have received financial support through the MYRIAD-EU project from the 10.13039/501100007601European Union’s Horizon 2020 research and innovation program (grant agreement no. 101003276). S.P. has received financial support through the EarlyWarning4IGAD project (grant agreement: UNDRR/GR/2023/027) and the EW4IGAD III project (grant agreement: UNDRR/GR/2024/045) funded by the UN Office for Disaster Risk Reduction (UNDRR). A.M. has received support from the PerfectSTORM ERC Grant Project (number: ERC-2020-StG-948601). The authors would like to thank Maarten de Vries for the contribution to the design and visualization of Figure 3 and the graphical abstract.

## Author contributions

T.T. and S.P. contributed equally and conceptualized the research, undertook the review/modeling, and led the analysis and investigation. A.M. and M.v.d.H. contributed to the conceptualization of the research and analysis. T.T. and S.P. wrote the first draft of the manuscript, which was edited and reviewed by A.M., M.v.d.H., L.T., M.R., and S.T.

## Declaration of interests

The authors declare no competing interests.

## STAR★Methods

### Key resources table

**Table:** 

REAGENT or RESOURCE	SOURCE	IDENTIFIER
**Software and algorithms**		

Python (version: 3.12)	The Python Software Foundation	RRID: SCR_008394, https://www.python.org/
spaCy (Natural Language Processing library)	Explosion AI	https://spacy.io/
Hugging Face (AI model repository and libraries)	Hugging Face Inc.	https://huggingface.co/
geopy (Geocoding library)	GeoPy contributors	https://github.com/geopy/geopy
Scopus (Bibliographic database)	Elsevier	https://www.scopus.com/
Microsoft Excel	Microsoft Corporation	RRID: SCR_016137, https://www.microsoft.com/

### Method details

We conducted a systematic literature review to gather descriptive statistics on the use of AI for early warning systems (EWS) and synthesised its role across the four pillars of EW4ALL using the pool of papers. In order to do so, we first developed search queries using Scopus (on title, abstract and keywords) incorporating terms related to EWS, AI (including ‘artificial intelligence’, ‘machine learning’, and ‘deep learning’), natural hazards, and risk, with some queries also linked to specific pillars (see literature selection). Subsequently, we screened the papers for relevance using a four-eye principle and gathered those that met our criteria of relevance to the pillars (see screening process). Next, we classified the entries, extracted relevant metadata, and employed NLP techniques to assist us to detect key terms. We have validated everything manually afterward (see review process). This process allowed us to compile a comprehensive dataset for our review, following the PRISMA 2020 guidelines where applicable.

#### Literature selection

For selection of relevant literature, we used Scopus and included criteria encompassing EWS, AI, and risk-related terminology. We divided the literature search among the pillars of EW4ALL and various categories (see Table 2). Each search query included one of the AI-related terms and ‘early warning’ for EWS, except in the case of single-hazard queries, where we used ‘early warning system’, and was applied to the title, abstract and keywords. For risk-related terminology, we selected the criteria listed in column 4 of Table 2 which resulted in a total of 1344 unique papers, and 1187 after excluding non-English written papers. The Table summarises the search criteria for the selection process of the systematic review in which each row represents a search criterion.

**Table:** Search criteria for the selection process of the systematic review in which each row represents a search criterion

EW4ALL pillar	Search terms^a^	Exclusion criteria
Pillar I risk knowledge	multi-hazard, multi-disaster, multi-risk, compound hazard, compound disaster, compound risk, impact based, exposure, vulnerability	socio-economic events
Pillar I risk knowledge or Pillar II forecasting, analysis, monitoring	hazard, disaster, flood, drought, heatwave, wildfire, earthquake, coldwave, landslide, avalanche, storm, cyclone, typhoon, hurricane, tsunami, volcano.	biohazards, socio-economic shocks, non-hydro- and geo-hazards
Pillar III warning dissemination and communication	dissemination, communication	urban digitalization, smart cities, health related studies
Pillar IV response and preparedness	preparedness, response, early action, anticipatory action, emergency response	military response, conflict response, conflict preparedness

a Note that all searches include the search terms on AI (“Artificial Intelligence” OR “AI” OR “Machine learning” OR “Deep learning”) and “early warning,” except the second row with individual hazards for which “early warning system” was used.

#### Screening process

For each entry, we employed the four-eye principle and screened (title, abstract, keywords) if the entries are addressing AI and EWS within the context of natural hazards, disasters, and risk. Here, we excluded entries related to biological hazards. To differentiate between Pillar I and Pillar II, we categorised entries based on whether they contributed to the body of knowledge on EWS (Pillar I) or provided a practical implementation of hazard monitoring and detection (Pillar II). This resulted in 324 papers for the review process.

#### Review process

For each of the 324 selected papers, we extracted key information as meta data that includes the AI concept employed (i.e., classical ML, NN, and NLP), the specific AI method, the geographical location, and the hazard type. We focused on three AI categories, i) classical machine learning, ii) neural networks, and iii) natural language processes and assigned each paper to the category it most closely aligned with. We used Natural Language Processing (NLP) tools to assist us in the review process for specific parts and tasks of the review process.^159^ We employed ‘spaCy’ for tokenisation and named entity recognition to extract locations and models from predefined lists, and a fine-tuned transformer model from the Hugging Face library to classify text into specific hazard types. This NLP-generated metadata served as a preliminary dataset that was subsequently manually verified, corrected and further filled in by the research team. Each entry was thoroughly reviewed for fitness with 100% human oversight of all extracted information, and the metadata was further filled in and updated accordingly to ensure accuracy. In addition, for papers that are mentioning specific geographical locations (*n* = 158), we manually classified each study based on implementation status: ‘research-based’ (AI methods applied to case study data with no operational deployment), ‘prototype or implemented’ (AI systems developed and tested but not yet operationally integrated or AI methods integrated into existing operational EWS).

### Quantification and statistical analysis

We used the geopy package in Python to extract latitude and longitude data for the case studies locations and generated descriptive plots for Figure 1. These plots illustrate the distribution of papers across different hazard types, pillars, and other relevant categories.

## Supplemental information

Data S1. Dataset of reviewed papers and associated metadata
